# Associations between fish intake and the metabolic syndrome and its components among middle-aged men and women: the Hordaland Health Study

**DOI:** 10.1080/16546628.2017.1347479

**Published:** 2017-07-13

**Authors:** Therese Karlsson, Hanne Rosendahl-Riise, Jutta Dierkes, Christian A Drevon, Grethe S Tell, Ottar Nygård

**Affiliations:** ^a^ Department of Clinical Science, Faculty of Medicine and Dentistry, University of Bergen, Bergen, Norway; ^b^ Department of Clinical Medicine, Faculty of Medicine and Dentistry, University of Bergen, Bergen, Norway; ^c^ Department of Nutrition, Institute of Basic Medical Sciences, Faculty of Medicine, University of Oslo, Oslo, Norway; ^d^ Department of Global Public Health and Primary Care, University of Bergen, Bergen, Norway; ^e^ Department of Health Registries, Norwegian Institute of Public Health, Oslo, Norway; ^f^ Department of Heart Disease, Haukeland University Hospital, Bergen, Norway

**Keywords:** Diet, triglycerides, HDL cholesterol, fatty fish, lean fish, metabolic syndrome

## Abstract

In epidemiologic studies, the relationship between fish consumption and the metabolic syndrome (MetS) have been inconclusive and sex differences reported. The aim was to investigate associations between fish intake and the MetS in a cross-sectional study of men and women. Fish intake, waist circumference, triglycerides (TG), HDL-C, glucose and blood pressure were assessed among 2874 men and women (46–49 y) in the Hordaland Health Study (1997–1999). Fatty fish intake was inversely associated with TG in men only; mean difference in TG between highest and lowest quartile of fatty fish intake was –0.33 mmol/L (95% CI: –0.51, –0.15). Lean fish intake was inversely associated with TG in women only; mean difference in TG between highest and lowest quartile of lean fish intake was –0.23 mmol/L (95% CI: –0.34, –0.11). Fatty fish intake was positively associated with serum HDL-C in both men and women. Total fish intake was inversely associated with MetS; adjusted OR 0.75 (95% CI 0.57, 0.97). Higher fish intake was associated with lower odds of having MetS possibly driven by associations of higher fish intake with lower TG and higher HDL-C. The findings of differential associations by sex needs to be confirmed and possible biologic mechanisms explored.

## Introduction

The metabolic syndrome (MetS) is a cluster of risk factors related to increased risk of cardiovascular disease and type 2 diabetes mellitus. Several diagnostic criteria exist, but all tend to agree on the inclusion of abdominal obesity (elevated waist circumference (WC)), hyperglycemia, elevated triglycerides (TG), low levels of high density lipoprotein cholesterol (HDL-C), and hypertension [1]. The prevalence of MetS is dependent on definition used and population studied. Prevalence estimates differ across sex, age, and ethnicity, as well as lifestyle habits and socioeconomic status [1]. In a population-based survey in Norway, the estimated prevalence of MetS in 1995–1997 was 27.6 and 21.8% in men and women (40–49 years), respectively [2]. The prevalence of the different MetS components was, except for central obesity, higher in men than women [2]. A healthy lifestyle, including diet and physical activity, is important for preventing and treating components of MetS [1].

Supplementation with the omega-3 (n-3) long-chain polyunsaturated fatty acids (LC-PUFA) eicosapentaenoic acid (EPA) and docosahexaenoic acid (DHA) reduces circulating TG and may have a small effect on increasing HDL-C [3]. Fish is the main dietary source of EPA and DHA but habitual fish consumption generally provides considerably lower amounts of these fatty acids than usually distributed in studies using n-3 LC-PUFA supplementation. Moreover, other nutrients in fish besides n-3 LC-PUFA may also affect components of MetS. Fish consumption contributes to dietary intake of selenium, vitamin D, protein, choline, and vitamin B_12_ and it is therefore important to explore not only specific nutrients but fish consumed.

In epidemiologic studies, the relationship between fish consumption and prevalence of MetS has shown either no association [4,5] or an inverse association [6,7]. Higher fish intake has been associated with lower TG and higher HDL-C [6,8,9]. However, most epidemiologic studies have focused on the association of total fish intake whereas very few have investigated associations of different types of fish consumed and MetS [9,10], which would provide additional information. An investigation including different types of fish intake requires that overall fish consumption is high enough to create sub-groups of fish intake. Fish intake in Norway is among the highest in Europe and a Norwegian cohort should therefore be well suited for studying sub-groups of fish [11]. Furthermore, one prospective study and one cross-sectional study relating fish intake with MetS reported an inverse association in men but no association in women [12,13]. Overall, considering sex differences in studies of diet and MetS is of importance. The aim of the current study was to investigate the association between intake of fish (total fish consumption as well as type of fish consumed) and MetS and its components in middle-aged men and women with habitually high fish consumption. Furthermore, possible effect modification by sex was explored.

## Subjects and methods

### Study population

The current investigation is a cross-sectional study among participants from the Hordaland Health Study (HUSK). HUSK was conducted during 1997–1999 as collaboration between the University of Bergen, the National Health Screening Service (now the Norwegian Institute of Public Health), and local health services. Extensive information of the study can be found at http://husk.b.uib.no. All participants signed an informed consent. The study protocol was in accordance with principles of the Declaration of Helsinki and the study was approved by the Regional Committee for Medical and Health Research Ethics.

In the current study, 3723 participants born 1950–1951 (age 46–49 years) who answered a food frequency questionnaire (FFQ) were included. Subjects who reported a very low (<3000 kJ/day for women and <3300 kJ/day for men) or high (>15,000 kJ/day for women and >17,500 kJ/day for men) energy intake were excluded (*n* = 78). In addition, patients with missing measures of MetS components or high-sensitivity C-reactive protein (CRP) (*n* = 165) were excluded leaving a total of 1225 men and 1649 women for the current analysis.

### Dietary assessment

A 169-item semi-quantitative FFQ developed at the Department of Nutrition, University of Oslo, Norway was used to estimate habitual dietary intake during the last year [14–16]. The FFQ was handed out on the day of the health examination, filled out at home, and returned by mail to the HUSK project center. Daily food (grams per day) and nutrient intakes (including supplements) were calculated using a database and software system developed at the Department of Nutrition, University of Oslo (Kostberegningssystem, version 3.2; University of Oslo, Norway). The food database was mainly based on the Norwegian food composition table, with some additional foods [17].

Items in the FFQ related to fish consumption included fish as spread (sandwich meals are common in Norway) or fish as part of main meals. Questions related to fish as spread were: How many slices of bread with the following spread do you eat per week; tinned mackerel in tomato paste or smoked mackerel; sardines; pickled herring; anchovies or similar fish; salmon or trout; and sandwich caviar. These questions included 11 frequency categories which ranged from 0 to ≥36 slices/week. Questions on fish intake as part of main meals included the following: fish cakes, fish pudding and fish balls (fish mixed with milk, flour and/or egg); fish fingers; boiled cod, coalfish and haddock; fried cod, coalfish and haddock; fresh, salt-cured or smoked herring; fresh or smoked mackerel; salmon or trout (both wild and farmed); and fish stew, fish soup and fish au gratin. The questions were two-fold and included nine frequency categories (0 to ≥9 times/month) and five portion size categories (piece, fillet, slice, or household measures depending on question). In the current analysis, the following categories of fish intake were constructed: ‘total fish’ (lean, fatty, unspecified fish as part of main meal, fish products, and fish as spread), ‘lean fish’ (cod, coalfish, or haddock as part of main meal), ‘fatty fish’ (mackerel, herring, trout, or salmon as part of main meal and fish as spread which included mackerel, salmon, trout, sardines, pickled herring, or sandwich caviar), and ‘fish products’ (fish fingers, fish pudding, fish cakes, etc.). For daily total marine n-3 LC-PUFA intake (diet and supplements), we used the sum of EPA, docosapentaenoic acid (DPA), and DHA. Participants reporting use of supplemental cod liver oil or fish oil were defined as users of such. Adjustment for total energy intake was performed using the multivariate nutrient density method, either as g/1000 kcal (foods) or as percent of total energy intake (macronutrients) [18].

### MetS definition

MetS was defined by criteria from the Joint Interim Societies using population specific cut-off for WC [19]. The cut-offs were as follow: WC ≥94 cm in men and ≥80 cm in women (Europid population); TG ≥1.7 mmol/L; HDL-C < 1.0 mmol/L in men and <1.3 mmol/L in women; elevated systolic blood pressure (SBP) ≥130 and/or diastolic blood pressure (DBP) ≥85 mmHg; elevated fasting glucose ≥5.5 mmol/L (serum glucose in this study was non-fasting). The presence of any three of these five factors constitutes a diagnosis of MetS.

### Biochemical data

Non-fasting blood samples were collected. Serum samples of total cholesterol, HDL-C, TG, and glucose were analyzed within 7 days at the department of Clinical Chemistry, Ullevål University Hospital, Oslo, using enzymatic methods with reagents from Boehringer Mannheim (Roche, Basel, Switzerland). Non-HDL-C was calculated as the difference between total cholesterol and HDL-C. Cotinine (biomarker of recent nicotine use) and CRP were measured in EDTA plasma stored at −80°C until analyzed at Bevital A/S (www.bevital.no) by LC/MS/MS and MALDI-TOF MS, respectively.

### Clinical data

Participants had a brief health examination including measurement of blood pressure, height, weight, and WC. After a 10 min seated rest, SBP and DBP were measured three times and the mean value of the second and third measurements was used (Dinamap 845 XT equipment (Criticon). In a sub-sample, body composition was measured by dual energy X-ray absorptiometry (Expert-XL; Lunar Company Inc., Madison, US). Information on educational level, medication use, smoking, and physical activity was collected through self-administered questionnaires. Participants answered two questions of leisure time physical activity referring to heavy physical activity (sweating and getting out of breath) or light physical activity (e.g. walking, gardening, housework with no sweating, or getting out of breath) in the past year (none, < 1 h/wk, 1–2 h/wk, or ≥3 h/wk). As previously described [20], categories for light physical activity were replaced with a value of 0 (none), 0.25 (<1 h/wk), 0.5 (1–2 h/wk), or 1.0 (≥3 h/wk) and for hard physical activity with 0 (none), 0.5 (<1 h/wk), 1.0 (1–2 h/wk), or 2.0 (≥3 h/wk). The sum of these scores was calculated and used in multivariate models. Current smokers were identified as participants having cotinine concentrations ≥85 nmol/L. In case of missing cotinine measures (*n* = 30) self-reported smoking status was used.

### Statistical analyses

Characteristics and daily dietary intake variables were summarized using means ± SD or medians (5th, 95th percentiles) for continuous variables and proportions for categorical variables. Baseline characteristics and dietary intake across quartiles of total fish intake were evaluated by using linear regression for continuous variables, logistic regression for binary variables, and Pearson’s Chi-square test for categorical variables. Differences between men and women were assessed by the Mann–Whitney *U* or Fisher’s exact tests.

Intake of fish was categorized into quartiles based on the total population with the lowest quartile as reference. Associations between intake of fish and components of MetS were assessed using multiple linear regression analysis. Fish intake variables were represented in the multiple linear regression models with indicator variables for each of the three non-reference quartiles (quartiles 2–4). Associations between intake of fish and prevalence of MetS were evaluated using logistic regression. In the multiple linear and logistic regression analyses, missing data (educational level and physical activity) were accounted for using multiple imputations. Confounders considered were age, sex, smoking, physical activity, educational level, estrogen use (women only) and dietary factors. Analyses were performed excluding individuals currently using antihypertensive medications. To explore any modifying effects by sex, analyses were stratified and interactions between fish intake and gender were tested by adding interaction product terms (fish intake quartiles*sex) into otherwise identical regression models. Due to non-fasting blood samples, sensitivity analyses of associations between fish intake and MetS were performed using an alternative cut-off of ≥6.0 mmol/L for defining elevated glucose. Statistical analyses were performed with SPSS for Windows, version 22 (IBM, NY, USA). A two-sided *p*-value <0.05 was considered statistically significant.

## Results

### Characteristics and dietary intake

Reported energy-adjusted total fish intake and absolute total fish intake were (mean ± SD) 34.4 ± 19.1 g/1000 kcal/d and 72.9 ± 46.0 g/d, respectively. Non-consumption (0 g/day) of total fish, lean fish, fatty fish, and fish products was reported by 0.6, 6.1, 8.9, and 6.0%, respectively. Compared with women, men were more likely to use lipid-modulating or anti-hyperglycemic drugs, having higher level of education, and to be overweight or obese (Table 1). Mean concentrations of TG, total cholesterol, CRP, SBP, and DBP were higher in men compared with women. Mean concentrations of HDL-C and body fat mass were higher in women than men (Table 1).

**Table 1.** Characteristics of 2874 men and women (46–49 years) in the Hordaland Health Study.

	Total *n* = 2874	Men *n* = 1225	Women *n* = 1649	*p*
Current smokers	35.9	35.8	36.0	0.94
Anti-hyperglycemic drugs	0.6	0.9	0.3	0.04
Antihypertensive drugs	5.3	5.4	5.2	0.80
Lipid-modulating drugs	2.2	3.3	1.3	<0.001
Estrogen therapy	18.3	NA	18.3	NA
Metabolic syndrome	30.0	37.3	24.6	<0.001
Educational level				<0.001
Primary school <10 y	19.4	15.7	22.2	
A-levels/high school	42.5	41.6	43.1	
College/University	38.1	42.7	34.7	
Hard physical activity				<0.001
None	25.9	28.9	21.8	
<1 h/wk	28.2	26.1	31.1	
1–2 h/wk	31.6	32.6	30.4	
≥3 h/wk	14.2	12.4	16.6	
BMI (kg/m^2^)				<0.001
<24.9	51.0	38.5	60.3	
25.0–29.9	38.6	50.1	30.0	
≥30.0	12.0	11.3	9.8	
Waist circumference (cm)	85.5 ± 11.6	92.7 ± 8.9	80.2 ± 10.4	<0.001
Body fat mass (%)	32.0 ± 9.6	24.6 ± 7.4	36.6 ± 7.7	<0.001
Systolic blood pressure (mmHg)	127 ± 15	131 ± 14	124 ± 15	<0.001
Diastolic blood pressure (mmHg)	75 ± 11	78 ± 9.9	72 ± 10	<0.001
Serum triglycerides (mmol/L)	1.70 ± 1.09	2.06 ± 1.23	1.43 ± 0.89	<0.001
Serum total cholesterol (mmol/L)	5.73 ± 0.95	5.83 ± 0.98	5.65 ± 0.92	<0.001
Serum HDL-C (mmol/L)	1.33 ± 0.37	1.15 ± 0.30	1.45 ± 0.36	<0.001
Serum non-HDL-C (mmol/L)	4.40 ± 1.02	4.68 ± 1.02	4.19 ± 0.97	<0.001
Serum glucose (mmol/L)	5.2 ± 1.0	5.3 ± 1.1	5.1 ± 0.9	<0.001
C-reactive protein (mg/L), median (IQR)	1.07 (2.11)	1.12 (2.07)	1.01 (2.14)	0.04

Values represent percentages and means ± SD. Missing data: education *n *= 26, physical activity *n* = 112 and body fat mass *n* = 441. *P* values for differences between men and women were calculated using Mann–Whitney *U* test or Fisher’s exact test for continuous and categorical variables, respectively. Blood sampling in the Hordaland Health Study was non-fasting.

A higher proportion of men than women used supplemental cod liver oil, 38.0% versus 33.4%, respectively (Table 2). Compared with men, women reported higher intakes of fiber, fruit and berries, and vegetables. A higher proportion of women compared with men reported consuming no alcohol. Energy-adjusted intake of total fish and types of fish (g/1000 kcal) was similar in men and women with only slightly higher lean fish intake in women compared with men (Table 2). Reported absolute intake (not adjusted for total energy intake) of total fish and types of fish was higher in men compared with women (data not shown).

**Table 2.** Daily dietary intakes of 2874 men and women (46–49 years) in the Hordaland Health Study.

	Total *n *= 2874	Men *n* = 1225	Women *n* = 1649	*p*
Energy (kcal)	2131 ± 625	2474 ± 615	1876 ± 496	<0.001
Carbohydrate (E%)	49.9 ± 5.9	49.5 ± 5.7	50.2 ± 6.0	<0.01
Fiber (g/1000 kcal)	11.8 ± 3.1	10.8 ± 2.6	12.5 ± 3.2	<0.001
Protein (E%)	16.1 ± 2.3	15.8 ± 2.2	16.3 ± 2.4	<0.001
Total fat (E%)	32.0 ± 5.1	32.1 ± 5.0	31.8 ± 5.2	0.16
SFA (E%)	12.3 ± 2.3	12.2 ± 2.3	12.4 ± 2.4	<0.01
MUFA (E%)	10.2 ± 1.8	10.3 ± 1.8	10.1 ± 1.8	0.01
PUFA (E%)	6.9 ± 2.0	7.1 ± 2.0	6.8 ± 2.0	<0.001
n-3 PUFA (E%)*^a^*	1.2 ± 0.4	1.2 ± 0.4	1.2 ± 0.4	0.03
n-3 LC-PUFA (E%)*^b^*	0.4 ± 0.3	0.4 ± 0.3	0.4 ± 0.4	0.39
n-6 PUFA (E%)*^c^*	5.6 ± 1.8	5.8 ± 1.8	5.5 ± 1.7	<0.001
Alcohol*^d^* (%)				<0.001
None	15.6	9.2	20.3	
Low-moderate	72.7	79.9	67.3	
Moderate	8.8	6.9	10.2	
High	3.0	3.9	2.2	
Supplement use (%)				
Fish oil use	8.0	7.8	8.2	0.73
Cod liver oil use	35.4	38.0	33.4	0.01
Food intake				
Vegetables (g/1000 kcal)	103 ± 74.1	78.8 ± 60.1	122 ± 78.1	<0.001
Fruit and berries (g/1000 kcal)	119 ± 78.6	96.7 ± 64.2	136 ± 84.0	<0.001
Meat (g/1000 kcal)	56.6 ± 23.2	57.3 ± 23.0	56.0 ± 23.3	0.09
Dairy products (g/1000 kcal)	145 ± 6.9	157 ± 104	136 ± 101	<0.001
Total fish (g/1000 kcal)	34.4 ± 19.1	33.6 ± 19.0	35.0 ± 19.2	0.04
Fatty fish (g/1000 kcal)	10.2 ± 10.3	10.6 ± 10.6	9.8 ± 10.1	0.07
Lean fish (g/1000 kcal)	13.0 ± 11.0	12.2 ± 10.6	13.6 ± 11.3	<0.001
Fish products (g/1000 kcal)	8.0 ± 5.9	8.0 ± 5.7	8.0 ± 6.0	0.52

Values represent percentages and means ± SD. *P* values for differences between men and women were calculated using Mann–Whitney *U* test or Fisher’s exact test for continuous and categorical variables, respectively. E%, percent of total energy intake, LC-PUFA, long-chain polyunsaturated fatty acid; MUFA, monounsaturated fatty acid; n-3, omega-3; n-6, omega-6; SFA, saturated fatty acid.

*^a^* Sum of α-linolenic acid, eicosapentaenoic acid, docosapentaenoic acid, and docosahexaenoic acid.

*^b^* Sum of eicosapentaenoic acid, docosapentaenoic acid, and docosahexaenoic acid.

*^c^* Sum of linoleic acid and arachidonic acid.

*^d^*None: 0 g/day; Low-moderate: women 0.1–10 g/day, men 0.1–20 g/day; Moderate: women 10–20 g/d, men 20–30 g/d; High: women >20 g/day, men >30 g/day.

Men and women with higher consumption of total fish had a higher intake of protein, fiber, vegetables, fruit and berries, and meat but a slightly lower intake of carbohydrates and SFA. Participants with higher intake of total fish were more likely to use fish oil (Supplementary Tables 1 and 2). Characteristics across quartiles of energy-adjusted total fish intake in men and women were explored. In men, higher intake of total fish was positively associated with use of lipid-modulating medications, BMI, WC and body fat mass. There were no statistically significant associations between total fish intake and current smoking, use of antihypertensive or anti-hyperglycemic medications, physical activity, educational level, or circulating CRP in men (data not shown). In women, higher intake of total fish was associated with lower level of education, lower serum TG and higher HDL-C concentrations. There were no statistically significant associations between fish intake and current smoking, use of lipid-modulating, antihypertensive or anti-hyperglycemic medications, current estrogen therapy, physical activity, or circulating CRP in women (data not shown).

Blood measures were non-fasting and for 82.2% of the subjects time since last meal was less than 4 h. Serum glucose was inversely associated with time since last meal (*rho* = −0.22, *p* < 0.001). TG were weakly inversely associated with time since last meal (*rho* = −0.05, *p* < 0.01) whereas HDL-C was not significantly associated with time since last meal (*rho* = −0.01, *p* = 0.68).

### Associations between intake of fish and MetS components

#### Total cohort

There were no associations between fish intake and SBP, DBP, or serum glucose (Table 3). Excluding individuals using anti-hypertensive drugs did not affect the results. In the total cohort, intake of fatty fish was inversely associated with TG and positively associated with HDL-C. Intake of lean fish was inversely associated with WC and TG. Overall, additional adjustment for educational level, physical activity, and dietary intake did not alter the results materially. Associations between fish intake and WC were slightly attenuated in models controlling for educational level, physical activity and dietary intake but remained statistically significant. Consumption of fish products was not significantly associated with any of the MetS components.

**Table 3.** Mean differences in components of the metabolic syndrome by quartiles of daily fish intake in reference to quartile one in 2874 men and women (46–49 years).

	Quartiles of fish intake					
	2nd	3rd	4th	*p*_trend_ _Model 1_	*p*_trend_ _Model 2_	*p*_interaction_
**Total fish (g/1000 kcal)**	26.4 (22.1, 31.0)*^a^*	36.7 (32.2, 43.2)	54.5 (44.7, 94.5)			
Waist circumference (cm)	−0.19 (−0.71, 0.33)	−0.32 (−0.84, 0.20)	−0.65 (−1.17, −0.13)	0.01	0.04	0.54
Triglycerides (mmol/L)	−0.04 (−0.14, 0.07)	−0.10 (−0.20, 0.01)	−0.18 (−0.28, −0.08)	<0.001	<0.01	0.58
HDL-C (mmol/L)	0.03 (−0.00, 0.06)	0.04 (0.00, 0.07)	0.06 (0.02, 0.09)	<0.001	<0.01	0.35
Systolic blood pressure (mmHg)	−0.70 (−2.18, 0.78)	0.21 (−1.27, 1.69)	0.90 (−0.58, 2.38)	0.13	0.20	0.64
Diastolic blood pressure (mmHg)	−0.21 (−1.24, 0.83)	0.06 (−0.97, 1.10)	−0.16 (−1.20, 0.87)	0.90	0.71	0.87
Glucose (mmol/L)	0.01 (−0.12, 0.09)	−0.05 (−0.15, 0.06)	0.02 (−0.08, 0.13)	0.83	0.90	0.72
**Lean fish (g/1000 kcal)**	8.3 (5.8, 10.6)*^a^*	13.9 (11.2, 17.4)	23.9 (18.2, 48.4)			
Waist circumference (cm)	−0.28 (−0.80, 0.24)	−0.23 (−0.75, 0.29)	−0.75 (−1.27, −0.23)	<0.01	0.04	0.10
Triglycerides (mmol/L)	−0.04 (−0.14, 0.07)	−0.07 (−0.17, 0.03)	−0.11 (−0.22, −0.01)	0.03	0.03	0.05
HDL-C (mmol/L)	0.01 (−0.03, 0.04)	−0.01 (−0.04, 0.03)	0.00 (−0.03, 0.03)	0.79	0.63	0.15
Systolic blood pressure (mmHg)	−0.22 (−1.70, 1.26)	1.78 (0.30, 3.26)	0.58 (−0.90, 2.06)	0.12	0.19	0.29
Diastolic blood pressure (mmHg)	0.29 (−0.75, 1.32)	0.55 (−0.48, 1.58)	0.00 (−1.03, 1.04)	0.87	0.98	0.45
Glucose (mmol/L)	−0.06 (−0.17, 0.04)	0.04 (−0.06, 0.15)	−0.01 (−0.12, 0.09)	0.70	0.96	0.56
**Fatty fish (g/1000 kcal)**	5.2 (3.4, 7.3)*^a^*	10.4 (7.9, 13.5)	19.9 (14.2, 46.7)			
Waist circumference (cm)	−0.21 (−0.73, 0.31)	−0.66 (−1.18, −0.14)	−0.35 (−0.87, 0.18)	0.08	0.13	0.06
Triglycerides (mmol/L)	−0.08 (−0.18, 0.02)	−0.10 (−0.20, 0.00)	−0.18 (−0.28, −0.07)	<0.001	<0.01	0.02
HDL-C (mmol/L)	0.04 (0.01, 0.07)	0.06 (0.03, 0.09)	0.08 (0.05, 0.11)	<0.001	<0.001	0.73
Systolic blood pressure (mmHg)	0.46 (−1.02, 1.94)	0.02 (−1.47, 1.49)	1.26 (−0.22, 2.75)	0.16	0.26	0.47
Diastolic blood pressure (mmHg)	0.16 (−0.87, 1.20)	−0.54 (−1.57, 0.50)	0.47 (−0.57, 1.50)	0.68	0.94	0.92
Glucose (mmol/L)	−0.02 (−0.12, 0.09)	−0.01 (−0.11, 0.09)	0.01 (−0.09, 0.12)	0.77	0.98	0.24
**Fish products**	5.6 (4.0, 7.0)*^a^*	8.9 (7.3, 10.9)	14.4 (11.5, 25.1)			
Waist circumference (cm)	−0.19 (−0.72, 0.33)	−0.35 (−0.87, 0.17)	−0.31 (−0.83, 0.21)	0.19	0.25	0.87
Triglycerides (mmol/L)	0.01 (−0.09, 0.12)	−0.09 (−0.19, 0.02)	−0.02 (−0.12, 0.08)	0.32	0.37	0.87
HDL-C (mmol/L)	−0.01 (−0.04, 0.02)	0.00 (−0.03, 0.03)	0.00 (−0.03, 0.03)	0.81	0.59	0.43
Systolic blood pressure (mmHg)	0.73 (−0.76, 2.22)	1.05 (−0.44, 2.53)	0.64 (−0.84, 2.12)	0.35	0.30	0.05
Diastolic blood pressure (mmHg)	0.36 (−0.68, 1.40)	0.28 (−0.76, 1.32)	0.15 (−0.88, 1.19)	0.82	0.64	0.36
Glucose (mmol/L)	−0.07 (−0.17, 0.04)	−0.09 (−0.20, 0.01)	0.00 (−0.10, 0.11)	0.93	0.98	0.70

Multiple linear regression was performed with all independent variables included in the model simultaneously (Model 1: energy intake, sex, BMI, and smoking; Model 2: energy intake, sex, BMI, smoking, educational level, physical activity, alcohol consumption, fiber intake, and vegetable intake). The unstandardized B coefficients (95% CI) from Model 1 are presented. *P* for trend was calculated using quartiles as a continuous variable in otherwise identical multiple linear regression models. *P* for interaction was evaluated by including the product term fish intake quartiles*sex in multivariate Model 1. Blood sampling in the Hordaland Health Study was non-fasting.

*^a^* Median (5th, 95th percentiles), *n* = 718–719 per quartile.

#### In men and women

We observed a significant interaction of gender for the associations between fish intake and TG, both for lean fish (*p_int_* = 0.05) and fatty fish (*p_int_* = 0.02) (Table 3). In men, fatty fish was inversely associated with WC and TG whereas intake of lean fish was inversely associated with WC and TG in women (Table 4). Intake of fatty fish was positively associated with HDL-C in both men and women. In women, the association between fatty fish intake and HDL-C was attenuated in the fully adjusted model, the mean difference in serum HDL-C between highest and lowest quartile was 0.04 mmol/L (95% CI: −0.00, 0.09, *p* = 0.07). Further adjustment for use of estrogen did not materially affect the results and is not included in the fully adjusted models. In men, the association between total and fatty fish intake and WC was attenuated when adjusted for educational level, physical activity and dietary intake. Mean difference in WC between highest and lowest quartile of total fish intake was −0.68 cm (95% CI: −1.46, 0.10, *p* = 0.09). Mean difference in WC between highest and lowest quartile of fatty fish intake was −0.61 cm (95% CI: −1.38, 0.16, *p* = 0.12).

**Table 4.** Mean differences in waist circumference, triglycerides, and HDL-C by quartiles of fish intake in reference to quartile one in 1225 men and 1649 women (46–49 years).

	Quartiles of fish intake				
	2nd	3rd	4th	*p*_trend_ _Model 1_	*p*_trend_ _Model 2_
***Men***					
**Total fish (g/1000 kcal)**	26.4 (22.0, 30.9)*^a^*	36.2 (32.3, 43.2)	54.2 (44.7, 97.4)		
Waist circumference (cm)	−0.27 (−1.02, 0.49)	−0.49 (−1.26, 0.27)	−0.87 (−1.64, −0.10)	0.02	0.08
Triglycerides (mmol/L)	−0.08 (−0.26, 0.10)	−0.06 (−0.24, 0.13)	−0.21 (−0.39, −0.02)	0.05	0.06
HDL-C (mmol/L)	0.03 (−0.01, 0.08)	0.01 (−0.03, 0.06)	0.05 (0.00, 0.09)	0.10	0.14
**Lean fish (g/1000 kcal)**	8.4 (5.7, 10.6)*^a^*	13.8 (11.1, 17.5)	24.3 (18.2, 45.5)		
Waist circumference (cm)	0.05 (−0.70, 0.79)	−0.02 (−0.78, 0.73)	−0.57 (−1.34, 0.21)	0.18	0.27
Triglycerides (mmol/L)	0.04 (−0.14, 0.22)	−0.07 (−0.25, 0.11)	0.02 (−0.16, 0.21)	0.87	0.86
HDL-C (mmol/L)	−0.01 (−0.05, 0.04)	−0.05 (−0.09, 0.00)	−0.02 (−0.07, 0.02)	0.13	0.11
**Fatty fish (g/1000 kcal)**	5.3 (3.3, 7.3)*^a^*	10.5 (7.9, 13.5)	19.7 (14.2, 49.2)		
Waist circumference (cm)	−0.15 (−0.93, 0.63)	−1.19 (−1.97, −0.42)	−0.74 (−1.51, 0.02)	<0.01	0.03
Triglycerides (mmol/L)	−0.14 (−0.33, 0.05)	−0.23 (−0.42, −0.05)	−0.33 (−0.51, −0.15)	<0.001	<0.001
HDL-C (mmol/L)	0.04 (−0.01, 0.08)	0.05 (0.01, 0.10)	0.09 (0.04, 0.13)	<0.001	<0.001
***Women***					
**Total fish (g/1000 kcal)**	26.4 (22.1, 31.1)*^a^*	36.8 (32.1, 43.2)	55.4 (44.7, 94.4)		
Waist circumference (cm)	−0.14 (−0.85, 0.58)	−0.18 (−0.89, 0.53)	−0.50 (−1.20, 0.21)	0.18	0.23
Triglycerides (mmol/L)	−0.02 (−0.13, 0.10)	−0.13 (−0.25, −0.02)	−0.18 (−0.29, −0.07)	<0.001	<0.001
HDL-C (mmol/L)	0.03 (−0.02, 0.08)	0.05 (0.01, 0.10)	0.06 (0.02, 0.11)	<0.01	0.05
**Lean fish (g/1000 kcal)**	8.2 (5.8, 10.7)*^a^*	14.0 (11.2, 17.4)	23.6 (18.2, 49.3)		
Waist circumference (cm)	−0.53 (−1.26, 0.19)	−0.40 (−1.11, 0.31)	−0.91 (−1.16, −0.20)	0.02	0.03
Triglycerides (mmol/L)	−0.11 (−0.22, 0.01)	−0.08 (−0.19, 0.04)	−0.23 (−0.34, −0.11)	<0.001	<0.001
HDL-C (mmol/L)	0.02 (−0.03, 0.06)	0.02 (−0.03, 0.07)	0.02 (−0.03, 0.06)	0.48	0.60
**Fatty fish (g/1000 kcal)**	5.1 (3.4, 7.3)*^a^*	10.4 (7.9, 13.4)	20.0 (14.2, 43.8)		
Waist circumference (cm)	−0.26 (−0.95, 0.44)	−0.27 (−0.97, 0.43)	−0.04 (−0.75, 0.67)	0.89	0.92
Triglycerides (mmol/L)	−0.03 (−0.14, 0.08)	−0.00 (−0.11, 0.11)	−0.07 (−0.19, 0.04)	0.32	0.46
HDL-C (mmol/L)	0.05 (0.00, 0.09)	0.06 (0.02, 0.11)	0.07 (0.03, 0.12)	<0.01	0.07

Multiple linear regression was performed with all independent variables included in the model simultaneously (Model 1: energy intake, BMI, and smoking; Model 2: energy intake, BMI, smoking, educational level, physical activity, alcohol consumption, fiber intake, vegetable intake and use of fish oil and/or cod liver oil). The unstandardized B coefficients (95% CI) from Model 1 are presented. *P* for trend was calculated using quartiles as a continuous variable in otherwise identical multiple linear regression models. Blood sampling in the Hordaland Health Study was non-fasting.

*^a^*Median (5th, 95thpercentiles).

In post-hoc analysis, stratification on serum TG concentrations < or ≥1.7 mmol/L revealed an inverse association between fatty fish intake and serum TG in men with TG concentrations ≥1.7 mmol/L but not with TG concentrations <1.7 mmol/L (Supplementary Table 3). No such patterns were found in women.

### Association between fish intake and MetS

In the total cohort, 30% had MetS and the prevalence was higher in men compared with women (Table 1). The prevalence of the MetS components in the total cohort were as follows; elevated WC 46.3%, elevated TG 36.2%, reduced HDL-C 34.3%, elevated blood pressure (SBP and/or DBP) 41.4%, and elevated glucose (non-fasting) 21.5%. When using cut-off ≥6.0 mmol/L instead of ≥5.0 mmol/L for defining elevated glucose (non-fasting), the prevalence of elevated glucose and MetS was 13.2% and 27.3%, respectively.

Higher intakes of total and fatty fish were inversely associated with the MetS (Table 5). After adjustment for educational level, physical activity, and dietary intake, higher fatty fish intake was no longer significantly associated with MetS; fully adjusted (model 2) OR (95% CI) for highest vs. lowest quartile was 0.79 (0.61, 1.02), *p* = 0.08). Intake of fish products was not associated with having MetS. There were no statistically significant interactions between fish intake and sex besides a tendency towards an interaction between lean fish intake and sex (Table 5). In women, a high intake (fourth quartile) of lean fish was inversely associated with MetS; fully adjusted (Model 2) OR (95% CI) for highest vs. lowest quartile was 0.63 (0.44, 0.92), *p* = 0.02 (*p* for trend = 0.06). Lean fish intake was not associated with MetS in men. Sensitivity analyses of associations between fish intake and MetS, using cutoff ≥6.0 mmol/L for defining elevated glucose, showed similar estimates.

**Table 5.** Odds ratio (95% CIs) for metabolic syndrome prevalence per quartile of fish intake in 2874 men and women (46–49 years)*^a^.*

	Quartiles of fish intake						
	1st	2nd	3rd	4th	*p*_trend_ _Model 1_	*p*_trend_ _Model 2_	*p*_interaction_
Total fish	1.00	0.92 (0.72, 1.19)	0.85 (0.66, 1.09)	0.72 (0.56, 0.93)	<0.01	0.03	0.14
Lean fish	1.00	0.96 (0.74, 1.24)	1.08 (0.84, 1.39)	0.81 (0.63, 1.06)	0.25	0.38	0.08
Fatty fish	1.00	0.90 (0.70, 1.17)	0.86 (0.67, 1.12)	0.76 (0.59, 0.99)	0.04	0.08	0.22
Fish products	1.00	0.95 (0.74, 1.23)	0.86 (0.67, 1.12)	0.91 (0.70, 1.17)	0.34	0.39	0.39

*^a^*Logistic regression was performed with all independent variables included in the model simultaneously (Model 1: energy intake, sex, BMI, and smoking; Model 2: energy intake, sex, BMI, smoking, educational level, physical activity, alcohol consumption, fiber intake, and vegetable intake). Results are presented as OR (95% CIs). *p* for trend was calculated using quartiles as a continuous variable in otherwise identical logistic regression models.

## Discussion

### Main observations

In this cross-sectional study including middle-aged men and women, high fish intake was associated with lower circulating TG and higher HDL-C. Notably, there were sex differences showing differential associations between type of fish consumed and serum TG. Reported intake of fatty fish was inversely associated with TG in men only, whereas higher lean fish intake was inversely associated with TG in women. Fatty but not lean fish consumption was positively associated with HDL-C in both men and women. High intake of total fish was inversely associated with MetS without clear evidence of differences by sex or type of fish consumed.

### Fish intake and MetS

Associations of fish consumption with MetS and its components have been investigated in both cross-sectional and prospective cohort studies. The observed inverse association between intake of fish and circulating TG and the positive association with HDL-C in the current study is largely in accordance with other studies. However, in contrast to us, most of these studies investigated intake of total fish only. Examining type of fish consumed in addition to total fish adds information when exploring the fish–MetS relationship. In the current study, both fatty and lean fish intake was inversely associated with serum TG, however, with differential associations in men and women. In a prospective cohort among Korean men and women (40–69 years), daily total fish consumption in comparison to less than once a week was associated with lower incidence of high serum TG in men but not women [12]. In an Iranian cross-sectional study including only women, higher total fish intake (tertile three vs. tertile one) was inversely associated with hypertriglyceridemia and low HDL-C levels [6]. Moreover, in the CARDIA study including young American men and women (18–30 years), higher total non-fried fish consumption was associated with lower incidence of abnormal TG and HDL-C [8]. In a Norwegian cross-sectional study, fatty fish intake was inversely associated with serum TG in both men and women whereas lean fish intake was positively associated with HDL-C in men only [10]. In intervention trials, the groups receiving fatty fish have decreased circulating TG [21,22], whereas groups receiving lean fish have in some [23] but not all [24–26] decreased TG. However, type of fish, overall dietary intake, study population and choice of control diets differ considerably between the studies.

We observed an inverse association between intake of fish and WC; however, considering the small effect size and possible inaccuracies in measuring WC, our findings may be of limited clinical relevance [27]. In accordance with our findings, other epidemiologic studies also report no or only modest associations between fish intake and elevated WC [8,9,12] or an increase in WC over time [28]. Our findings of no significant associations between fish intake (neither total nor types of fish) and SBP, DBP, or glucose are in line with results from other epidemiologic studies showing no [8,9,12,29] or only modest associations [9].

### Possible mechanisms

Findings from the present study are biologically plausible. Fish, particularly fatty fish, is the major dietary source of the n-3 LC-PUFAs EPA and DHA, and the TG lowering effects of EPA and DHA are well documented [30,31]. The proposed mechanisms, although not fully understood, are decreased availability of free fatty acids, competitive inhibition of acyl-CoA:1,2-diacylglycerol acyltransferase, suppression of lipogenic genes, induction of fatty oxidation genes and increased lipolytic activity of lipoprotein lipase in extrahepatic tissues [3,32]. The effects of n-3 LC-PUFA on circulating HDL-C are less certain and usually smaller, and the evidence is inconsistent [3]. Historically, health benefits of increased fish consumption have been credited its content of n-3 LC-PUFA, but fish intake also contributes with considerable amounts of other nutrients such as protein, vitamin D, B-vitamins, iodine, and selenium. Protein from fish has been proposed, primarily based on studies in animals, to have a beneficial effect on lipid metabolism [33]. The evidence in humans is, however, limited, with two intervention studies reporting no effect of isolated cod protein on circulating TG and HDL-C [34,35]. Vitamin D status (25-hydroxyvitamin D) has been inversely associated with MetS and its components [36] but there is limited evidence from vitamin D supplementation trials on the effects on serum lipids [37,38]. Thus, the higher intake of n-3 LC-PUFA with higher fish consumption may well be the most important explanatory factor, but additional effects of other nutrients present in fish cannot be excluded and may explain the associations of lean fish intake.

Importantly, the intake of fish will replace other foods and depending on food replaced this will affect associations between fish consumption and MetS and its components. Fish usually replaces meat and higher meat consumption has been associated with increased risk of having MetS [7,39]. In addition, meals including fish may include other side dishes compared with meals including meat which would further affect overall dietary intake [40].

### Sex differences

We observed different associations for men and women regarding type of fish consumed in relation to serum TG. Fatty fish intake was inversely associated with TG in men but not women, whereas lean fish intake was inversely associated with TG in women only. To the best of our knowledge, only two cross-sectional studies in addition to ours, have evaluated lean and fatty fish intake separately in relation to TG by sex. In contrast to our observations, Torris et al. presented opposite associations in a population study in Northern Norway in 1994–1995 (the Tromsø 4 study) [9]. Higher intake of lean fish was associated with lower TG in men but not in women (models adjusted for age and total energy intake) [9]. The population in the Tromsø study is seemingly similar to ours; same country and time of observation, and with presumably similar fish consumption regarding type of fish [9]. However, the range of age for individuals included was larger (26–70 years) and adjustment for confounding factors as well as categorization of fish intake was different. On the other hand, in the following Tromsø 6 study with inclusion of individuals in 2007–2008 from the same geographical area, fatty and lean fish intake was associated with lower TG both in men and women (models were only adjusted for age) [10].

The sex differences are not easily explained. One possible explanation of the observed relationship between higher fatty fish intake and lower TG in men but not women could be the higher levels of TG and absolute fish intake (and thus n-3 LC-PUFA) in men. The magnitude of the TG reducing effects of EPA+DHA has been shown to be dose-dependent and higher baseline TG concentrations predicted a greater response to EPA+DHA [31]. Indicative of such a relation is the post-hoc analysis in the current study with associations between fatty fish intake and lower TG being present only in men with TG ≥1.7 mmol/L. Although intake of fish and thus n-3 LC-PUFA were relatively high, and the level of n-3 LC-PUFA intake in Norwegian women is higher compared to other populations, the possibility that the intake of n-3 LC-PUFA was too low to effect circulating TG in women cannot be excluded. In addition, hormone-dependent sex differences in lipid metabolism exist and if different biological responses to diet exist this could affect the results [41]. We had no information on hormonal status, and the menopausal status was not known for all the women. Adjusting for use of estrogens did not materially affect the results. Furthermore, the overall dietary pattern and lifestyle factors related to higher fish intake or type of fish may be different among men and women and could contribute to an overall better metabolic health. Although some dietary and lifestyle factors were accounted for in multivariate analysis, the complex relations of dietary intake and lifestyle factors with health outcomes are difficult to account for in observational studies.

### Strengths and limitations

The main strengths of the current study include its large sample size and the available data on clinical and lifestyle characteristics of the participants. Importantly, in addition to total fish intake, the current FFQ allowed us to also examine type of fish consumed.

There are limitations that should be highlighted. We appreciate the problems with measuring true dietary intake using self-reporting questionnaires. The FFQ used in the current study has previously been evaluated against dietary record and fatty acid composition in plasma phospholipids in other Norwegian populations [15,16]. Reported fish intake and n-3 LC-PUFA concentrations in plasma phospholipids was positively correlated (*r *= 0.37) [16]. Furthermore, reported fish intake in our study was similar to that of other Norwegian studies [11,42]. Thus, the FFQ used in the current study should estimate fish intake reasonably well, although the variation between individuals may be considerable and misclassification is possible [16]. The species of fish, preparation methods, seasonal variation, and possible contaminants of fish consumed could not be examined in the current study and assessment of these factors will provide additional information regarding fish–MetS relations. Blood measures in HUSK were non-fasting. HDL-C seems to be minimally changed to normal food intake whereas TG is affected by recent food intake [43]. In the current study, serum TG was only weakly (*rho* = 0.05, *p* < 0.01) inversely associated with time since last meal. In sensitivity analyses, using a higher cut-off for defining elevated glucose (≥6.0 instead of ≥5.0 mmol/L), associations between fish intake and MetS was not changed. Yet, MetS prevalence estimates should be interpreted with caution due to risk of misclassification and overestimation. Moreover, although several confounding factors were considered, residual confounding of unknown or imprecisely measured factors cannot be excluded.

## Conclusion

Fish intake was inversely associated with serum TG; intake of lean and fatty fish had differential associations in men and women. The observations of effect modification by sex and type of fish consumed suggest that sex differences may be important to explore in intervention studies with the final aim to individualize nutritional recommendations.

## Supplementary Material

Supplement_tables.pdf

## Supplemental material

The supplemental material for this article can be accessed here.
