# Supplementary material for: Associations between fish intake and the metabolic syndrome and its components among middle-aged men and women: the Hordaland Health Study
Source: Food Nutr Res. 2017 Jul 13;61(1):1347479. doi: 10.1080/16546628.2017.1347479 (PMC5510233; doi:10.1080/16546628.2017.1347479)
Supplement: Supplement_tables.pdf [file zfnr_a_1347479_sm9186.pdf]

Supplementary table 1 Daily dietary intake in 1225 men (46-49 years) by quartiles of total fish intake

	Quartiles of total fish intake				P trend
	**1st** n **= 323**	**2nd** n **= 313**	**3rd** n **= 299**	**4th** n **= 290**	
Energy (kcal)	2397 ± 614	2569 ± 594	2503 ± 594	2429 ± 647	0.77
Carbohydrate (E%)	50 8 ± 6.1	50.2 ± 5.4	48.9 ± 5.1	47.9 ± 5.8	<0.001
Fiber (g/1000 kcal)	10.6 ± 2.8	10.7 ± 2.4	10.7 ± 2.6	11.3 ± 2.7	<0.01
Protein (E%)	14.6 ± 2.1	15.3 ± 1.7	16.1 ± 1.8	17.4 ± 2.0	<0.001
Total fat (E%)	32.1 ± 5.4	31.9 ± 4.9	32.5 ± 4.7	32.1 ± 5.0	0.60
SFA (E%)	12.4 ± 2.5	12.2 ± 2.1	14.5 ± 2.2	11.8 ± 2.2	<0.01
MUFA (E%)	10.3 ± 2.0	10.2 ± 1.7	10.3 ± 1.5	10.4 ± 1.8	0.35
PUFA (E%)	7.0 ± 2.2	7.1 ± 2.1	7.2 ± 2.0	7.3 ± 1.8	0.04
n-3 (E%)	1.0 ± 0.4	1.1 ± 0.3	1.2 ± 0.3	1.4 ± 0.5	<0.001
n-3 LC-PUFA (E%)	0.2 ± 0.2	0.3 ± 0.2	0.4 ± 0.3	0.6 ± 0.4	<0.001
n-3 LC-PUFA (g)	0.6 ± 0.7	0.9 ± 0.7	1.1 ± 0.8	1.7 ± 1.2	<0.001
n-6 (E%)	5.8 ± 2.0	5.8 ± 1.8	5.8 ± 1.8	5.6 ± 1.6	0.21
Alcohol[a]					0.02
None	12.1	9.9	7.7	6.9	
Low-moderate	77.1	76.7	83.9	82.4	
Moderate	6.5	9.6	3.3	8.3	
High	4.3	3.8	5.0	2.4	
Vegetables (g/1000 kcal)	65.6 ± 61.7	73.7 ± 52.0	82.8 ± 57.7	94.8 ± 64.7	<0.001
Fruit and berries (g/1000 kcal)	91.1 ± 65.2	97.4 ± 62.5	92.0 ± 58.5	107 ± 69.3	0.01
Meat (g/1000 kcal)	55.0 ± 25.0	55.6 ± 22.3	59.1 ± 20.9	59.9 ± 23.1	<0.01
Dairy products (g/1000 kcal)	158 ± 115	160 ±102	155 ± 101	152 ± 97.8	0.43
Fish oil use	6.2	5.4	9.0	10.7	0.01
Cod liver oil use	37.2	39.0	38.1	37.9	0.90

Values represent percentages and means ± SD. *P* for trend was calculated using linear regression for continuous data and Pearson Chi-square test for categorical data. Dietary intake was adjusted for total energy intake using nutrient density method (g/1000 kcal or E%). E%, percent of total energy intake; MUFA, monounsaturated fatty acid; PUFA, polyunsaturated fatty acid; SFA, saturated fatty acid.

[a]None: 0 g/day; Low-moderate: 0.1-20 g/day; Moderate: 20-30 g/d; High: >30 g/day.

Supplementary table 2 Daily dietary intake in 1649 women (46-49 years) by quartiles of total fish intake

	Quartiles of total fish intake				P_{trend}
	**1st** *n* **= 395**	**2nd** *n* **= 406**	**3rd** *n* **= 420**	**4th** *n* **= 428**	
Energy (kcal)	1848 ± 539	1945 ± 506	1865 ± 461	1846 ± 473	0.39
Carbohydrate (E%)	52.0 ± 6.4	50.5 ± 5.6	50.3 ± 5.4	48.1 ± 6.0	<0.001
Fiber (g/1000 kcal)	11.9 ± 3.3	12.3 ± 3.1	12.8 ± 3.2	12.9 ± 3.2	<0.001
Protein (E%)	15.1 ± 2.2	15.6 ± 2.0	16.5 ± 2.0	18.0 ± 2.3	<0.001
Total fat (E%)	31.5 ± 5.8	32.1 ± 5.1	31.6 ± 5.1	32.0 ± 4.9	0.34
SFA (E%)	12.7 ± 2.9	12.6 ± 2.3	12.3 ± 2.3	12.2 ± 2.1	<0.001
MUFA (E%)	9.9 ± 1.9	10.1 ± 1.8	10.1 ± 1.8	10.2 ± 1.7	0.07
PUFA (E%)	6.5 ± 2.1	6.9 ± 1.9	6.7 ± 1.9	7.1 ± 1.9	<0.001
n-3 (E%)	1.0 ± 0.4	1.1 ± 0.4	1.2 ± 0.4	1.4 ± 0.5	<0.001
n-3 LC-PUFA (E%)	0.2 ± 0.3	0.3 ± 0.3	0.4 ± 0.3	0.6 ± 0.4	<0.001
n-3 LC-PUFA (g)	0.5 ± 0.6	0.7 ± 0.7	0.8 ± 0.7	1.2 ± 0.9	<0.001
n-6 (E%)	5.4 ± 1.9	5.7 ± 1.7	5.4 ± 1.6	5.5 ± 1.7	0.75
Alcohol[a]					0.04
None	26.1	17.0	18.6	19.6	
Low-moderate	63.8	69.7	70.5	65.0	
Moderate	8.1	10.6	9.0	13.1	
High	2.0	2.7	1.9	2.3	
Vegetables (g/1000 kcal)	99.4 ± 73.1	112 ± 70.6	131 ± 84.2	142 ± 76.7	<0.001
Fruit and berries (g/1000 kcal)	134 ± 96.3	139 ± 82.1	138 ± 76.6	134 ± 80.6	0.99
Meat (g/1000 kcal)	52.9 ± 24.0	55.6 ± 22.4	56.9 ± 22.3	58.4 ± 24.1	<0.001
Dairy products (g/1000 kcal)	144 ± 113	136 ± 94.1	141 ± 101	124 ± 94.2	0.01
Fish oil use	5.8	7.4	7.6	11.7	<0.01
Cod liver oil use	27.6	37.2	36.9	31.5	0.30

Values represent percentages and means ± SD. *P* for trend was calculated using linear regression for continuous data and Pearson Chi-square test for categorical data. Dietary intake was adjusted for total energy intake using nutrient density method (g/1000 kcal or E%). E%, percent of total energy intake; MUFA, monounsaturated fatty acid; PUFA, polyunsaturated fatty acid; SFA, saturated fatty acid.

[a]None: 0 g/day; Low-moderate: 0.1-10 g/day; Moderate: 10-20 g/d; High: >20 g/day.

Supplementary table 3 Mean differences in serum triglycerides by quartiles of fatty or lean fish intake in reference to quartile one in 1225 men and 1649 women (46-49 years) stratified on serum triglycerides

		Quartiles of fish intake			***P*** **trend**	***P*** **trend**
		**2nd**	**3rd**	**4th**	**Model 1**	**Model 2**
Men	*n*					
Fatty fish						
Triglycerides <1.7 mmol/L	579	0.06 (-0.01, 0.13)	0.01 (-0.06, 0.08)	0.00 (-0.07, 0.07)	0.54	0.51
Triglycerides ≥1.7 mmol/L	646	-0.01 (-0.28, 0.26)	-0.26 (-0.52, -0.00)	-0.31 (-0.57, -0.05)	<0.01	<0.01
Lean fish						
Triglycerides <1.7 mmol/L	579	-0.02 (-0.08, 0.04)	0.03 (-0.04, 0.09)	-0.02 (-0.09, 0.05)	0.99	0.99
Triglycerides ≥1.7 mmol/L	646	0.05 (-0.21, 0.30)	-0.13 (-0.39, 0.14)	0.03 (-0.24, 0.29)	0.82	0.91
Women						
Fatty fish						
Triglycerides <1.7 mmol/L	1254	0.00 (-0.04, 0.05)	0.01 (-0.04, 0.05)	-0.03 (-0.08, 0.02)	0.25	0.23
Triglycerides ≥1.7 mmol/L	395	-0.12 (-0.42, 0.19)	-0.02 (-0.33, 0.29)	0.06 (-0.27, 0.38)	0.64	0.53
Lean fish						
Triglycerides <1.7 mmol/L	1254	-0.00 (-0.05, 0.05)	-0.00 (-0.05, 0.05)	-0.05 (-0.10, -0.00)	0.03	0.06
Triglycerides ≥1.7 mmol/L	395	-0.09 (-0.40, 0.22)	-0.04 (-0.34, 0.26)	-0.17 (-0.48, 0.15)	0.38	0.40

Multiple linear regression was performed with all independent variables included in the model simultaneously (Model 1: energy intake, BMI, and smoking; Model 2: energy intake, BMI, smoking, educational level, physical activity, alcohol consumption, fiber intake, and vegetable intake). The unstandardized B coefficients (95% CI) from Model 1 are presented. *P* for trend was calculated using quartiles as a continuous variable in otherwise identical multiple linear regression models. Blood sampling in the Hordaland Health Study was non-fasting.
